# Catalog of the phylloxerids of the world (Hemiptera, Phylloxeridae)

**DOI:** 10.3897/zookeys.629.10709

**Published:** 2016-11-07

**Authors:** Colin Favret, Roger L. Blackman, Gary L. Miller, Benjamin Victor

**Affiliations:** 1 University of Montreal, Biodiversity Centre, 4101 rue Sherbrooke est, Montreal, Quebec, H1X 2B2 Canada; 2 The Natural History Museum, Department of Life Sciences, Cromwell Rd, London SW7 5BD, United Kingdom; 3 USDA-ARS, Systematic Entomology Laboratory, 10300 Baltimore Ave, Bldg. 005, BARC-West, Beltsville, MD 20705; 4 University of Montreal, Classical Studies Centre, 3774 rue Jean-Brillant, Montreal, Quebec, H3T 1P1 Canada

**Keywords:** Aphidomorpha, nomenclature, Phylloxera, Sternorrhyncha, taxonomy

## Abstract

A taxonomic and nomenclatural catalog of the phylloxerids (Hemiptera, Phylloxeridae) is presented. Six family-group names are listed, three being synonyms. Thirty-five genus-group names, of which six are subjectively valid, are presented with their type species, etymology, and grammatical gender. Ninety-four species-group names are listed, of which 73 are considered subjectively valid. This is the last group of Aphidomorpha to be catalogued, bringing the list of valid extant species to 5,218.

## Introduction

Phylloxeridae is a small family of Hemiptera, closely related to Adelgidae and Aphididae. Little is known of the biology of most of the family’s 69 species, although that of the economically important grape phylloxeran, *Daktulosphaira vitifoliae* (Fitch), has been studied in detail. Most species of phylloxerid feed on species of Juglandaceae or Fagaceae, with a large number forming galls on North American hickories (*Carya* spp.). Host alternation exists within the family (Stoetzel 1985) but it is either rare or understudied. Two fossil species are known, *Palaeophylloxera seilacheri* Heie and Peñalver 1999 and *Acanthochermes longirostris* Wegierek 2003, from the Miocene and Eocene, respectively.

Phylloxeridae is one of three extant families in the infraorder Aphidomorpha (Hemiptera, Sternorrhyncha) (Heie and Wegierek 2009). Whereas the Aphididae have been catalogued several times (Wilson and Vickery 1918, Eastop and Hille Ris Lambers 1976, Remaudière and Remaudière 1997) and the Adelgidae recently (Favret et al. 2015), the Phylloxeridae have not been comprehensively treated until now. Including the fossil taxa (Heie and Wegierek 2011), the entire infraorder has now been fully catalogued: 5,218 valid extant and 314 valid extinct species (Aphid.SpeciesFile.org).

In this catalog, we present six family-group, 35 genus-group, and 94 species-group names of extant phylloxerids. The family-group names include two valid subfamilies and two valid tribes and three subjective synonyms. The genus-group names include six valid names, 21 junior subjective synonyms, three junior objective synonyms, three junior homonyms, and two unavailable names. The species-group names include four subspecies (not including nominotypical subspecies), 14 subjective synonyms, one junior primary homonym, two nomina dubia, and four unavailable names.

The name Phylloxeridae in English is usually pronounced with the accent on the third syllable. However, the name of its type genus, *Phylloxera*, is often pronounced with an accent on the second. Because the *e* of *xērós* is an eta, the word made from it, once written in Roman letters and given Latin endings, must be considered to have a long *e*. The penultimate syllable of a Latin word must be accented when it contains such a long vowel and it is a fixed principle that the accentuation of Latin words is to be kept when they are borrowed into English. Therefore, strictly-speaking, only accentuation of the third syllable of *Phylloxera* is historically justified.

Russell (1975) described the complex history of the name of the grape phylloxeran, including the correct spelling of its generic name, *Daktulosphaira* Shimer 1866. Shimer also established *Dactylosphaera* (1867), probably meaning the latter to be an emended spelling of the former. The philological side of the alternate spellings can be stated briefly: k and c have both been used to transliterate classical Greek kappa, u and y to render upsilon, ai and ae the diphthong alpha+iota. C, y and ae were the preferred transliterations in classical Latin. K, u and ai are mostly used in linguistic circles that seek a more direct reflection of the phonetics of ancient Greek, bypassing the intermediary of Latin. Zoological nomenclature imposes Latin terminations, hence supposes Latinization of Greek (and other non-Latin) elements. *Dactylosphaera* is therefore the spelling more in the spirit of the system, although per ICZN Article 32.5.1 (International Commission on Zoological Nomenclature 1999), “incorrect transliteration or Latinization … are not to be considered inadvertent errors.” In addition to these two official spellings, other authors have used every possible combination of c/k, u/y and ae/ai to refer to the grape phylloxeran, giving *Dactulosphaera* (e.g., Kleeburg and Hummel 2001), *Dactulosphaira* (e.g., Fahrentrapp et al. 2015), *Dactylosphaira* (e.g., Alleweldt et al. 1991), *Daktulosphaera* (e.g., Loxdale 2008), *Daktylosphaera* (e.g., Tecchio et al. 2007), and *Daktylosphaira* (e.g., Torregrossa et al. 1997).

In any case, Shimer (1866, 1867) established different type species for *Daktulosphaira* and *Dactylosphaera*, thus the two spellings must be considered independent, available genus-group names (Wilson 1910). *Dactylosphaera globosa* Shimer 1867, one of a large number of North American hickory-feeding species, is the type species of its genus. As a consequence, *Dactylosphaera* has priority over all other generic names attributable to this distinct group. These include *Xerophylla*, described by Walsh later the same year (1867), *Euphylloxera* Del Guercio 1908, *Notabilia* Mordvilko 1909, *Paramoritziella* Grassi 1912, *Parapergandea* Börner 1930, *Pergandea* Börner 1908b, and *Troitzkya* Börner 1930. If we consider a key diagnostic character of the hickory-feeding species, the lack of abdominal spiracles, we can add *Acanthaphis* Del Guercio 1908 and *Moritziella* Börner 1908b to the list. It will require a thorough taxonomic revision of the phylloxerid family to correctly assign the various species, many of which are hardly known, to any of these listed generic names. Given this fact, the unfortunate history and spelling problems associated with *Dactylosphaera* and *Daktulosphaira*, and the fact that the identity and validity of the type species of *Dactylosphaera* may be questionable (Russell 1975), we have chosen to present a conservative classification, retaining the majority of species within the genus *Phylloxera* Boyer de Fonscolombe 1834. At some future date when more information is available, it may in particular be necessary to formalize a distinction between the Palearctic species (abdominal spiracular plates present) and the Nearctic species, species that typically form galls on hickories (abdominal spiracular plates absent, where known). As with the Catalog of Adelgidae (Favret et al. 2015), it is our hope that the present Catalog of Phylloxeridae will serve to stimulate interest and research on this insect group.

Also as with other recent catalogs of groups of Aphidomorpha, the etymology and grammatical gender of genus-group names has been included (Favret et al. 2008, 2009, 2015, Cortés Gabaudan et al. 2011, Nieto Nafría et al. 2011). Where original descriptions are listed with two page numbers, the first refers to a nomenclaturally valid diagnosis (e.g., a dichotomous key) and the second refers to the formal description. Valid names are listed in bold and synonyms preceded by ‘=’. The rank-specific endings of family-group synonyms are replaced by ‘–’. Species-group names are presented according to their current generic placement, their original generic placements in parentheses. An alphabetical index following the catalog provides the current placement of each name. Future updates will be published on Aphid Species File (Aphid.SpeciesFile.org).

## Catalogue

**PHYLLOXERIDAE**Herrich-Schaeffer 1854

Subfamily **PHYLLOXERINAE**Herrich-Schaeffer 1854

Tribe **ACANTHOCHERMESINI**Börner 1913: 667

Original spelling. Acanthochermesini

Type genus. *Acanthochermes*Kollar 1848

***ACANTHOCHERMES***Kollar 1848: 191

Type species. *Acanthochermes quercus*Kollar, 1848, by original monotypy

Etymology. Greek ákantha ‘thorn’ + *Chermes* [Hemiptera]

Gender. Masculine

***quercus***Kollar 1848: 191 (*Acanthochermes*)

=*balbianii* (Lichtenstein 1874a: 782) (*Phylloxera*)

***similiquercus***Jiang et al. 2009: 44,45 (*Acanthochermes*)

Tribe **PHYLLOXERINI**Herrich-Schaeffer 1854:VII

Original spelling. Phylloxeriden

Type genus. *Phylloxera*Boyer de Fonscolombe, 1834

=DACTYLOSPHAER– Shimer 1867: 2

Original spelling. Dactylosphaeridae

Type genus. *Dactylosphaera*Shimer, 1867

=MORITZIELL– Börner 1908b: 607

Original spelling. Moritziellini

Type genus. *Moritziella*Börner 1908b

=VACUN– Herrich-Schaeffer 1854:VII

Original spelling. Vacuniden

Type genus. *Vacuna*von Heyden 1837

***APHANOSTIGMA***Börner 1909b: 61

Type species. *Phylloxera piri*Cholodkovsky 1904, by original monotypy

Etymology. Greek aphanḗs ‘invisible’ + -o + Greek stigma ‘spot’ [pterostigma]

Gender. Neuter

=*CINACIUM*Kishida 1924: 473

Type species. *Cinacium iaksuiense*Kichida 1924, by original monotypy

Etymology. Japanese Kinako ‘soybean flour’ + -ium

Gender. Neuter

***iaksuiense*** (Kishida 1924: 473) (*Cinacium*)

***piri*** (Cholodkovsky 1904: 119) (*Phylloxera*)

***DAKTULOSPHAIRA***Shimer 1866: 365

Type species. *Pemphigus vitifoliae*Fitch 1855, by original monotypy

Etymology. Greek dáktylos ‘finger’ + Greek sphaîra ‘ball’

Gender. Feminine

=*PERITYMBIA*Westwood 1869: 109

Type species. *Peritymbia vitisana*Westwood 1869, by original monotypy

Etymology. Greek perí ‘around’ + Greek týmbos ‘tomb’ [“tomb-like gall”]

Gender. Feminine

Note. Some references cite Westwood 1867: 6, but this is a note referencing an oral presentation that was never published (Westwood 1877:xlvii).

=*RHIZAPHIS* Planchon in Bazille et al. 1868: 336

Type species. *Rhizaphis vastatrix* Planchon 1868, by original monotypy

Etymology. Greek ríza ‘root’ + *Aphis* [Hemiptera: Aphididae]

Gender. Feminine

=*RHIZOCERA*Despeissis 1896: 14

Type species. None

Etymology. Greek ríza ‘root’ + Greek xērós ‘dry’ [“root drier” per Despeissis 1896, but note, Latin cēra ‘wax’]

Gender. Feminine

Note. Unavailable, not proposed as a valid name. Often misattributed to Kirk 1897: 8.

=*VITEUS*Shimer 1867: 6

Type species. *Pemphigus vitifoliae*Fitch 1855, by original monotypy

Etymology. Latin ‘of or pertaining to the vine’

Gender. Masculine

Note. Junior objective synonym of *Daktulosphaira*Shimer 1866

=*XERAMPELUS*Del Guercio 1900: 77,80

Type species. *Rhizaphis vastatrix* Planchon 1868, by original monotypy

Etymology. Greek xērós ‘dry’ + Greek ámpelos ‘vine’

Gender. Masculine

Note. Junior objective synonym of *Rhizaphis* Planchon 1868

***vitifoliae*** (Fitch 1855: 862) (*Pemphigus*)

=*pemphigoides* (Donnadieu 1887: 1246) (*Phylloxera*)

=*pervastatrix* (Börner 1910: 4) (subspecies of *Peritymbia vitifoliae* (Fitch))

=*vastatrix* (Planchon in Bazille et al. 1868: 336) (*Rhizaphis*)

=*vitisana* (Westwood 1869: 109) (*Peritymbia*)

=*vitis viniferae* (Theobald 1914: 337) (*Phylloxera*) nomen nudum

=*vulpinae* (Börner 1952: 213) (subspecies of *Viteus vitifoliae* (Fitch))

***FOAIELLA***Börner 1909b: 61

Type species. *Phylloxera danesii*Grassi and Foà 1907, inherited from replaced name

Etymology. (Anna) Foà [Italian entomologist] + -i + ella [diminutive suffix]

Gender. Feminine

Note. Replacement name for *Boerneria*Grassi and Foà 1908. Described as subgenus of *Peritymbia*Westwood 1869

=*BOERNERIA*Grassi and Foà 1908: 685

Type species. *Phylloxera danesii*Grassi and Foà 1907, by original monotypy

Etymology. (Carl) Börner [German entomologist] + -ia

Gender. Feminine

Note. Junior homonym of *Boerneria*Willem 1902: 4 (Collembola) and *Boerneria*Axelson 1902: 101 (Collembola). Replaced by *Foaiella*Börner 1909b

***danesii*** (Grassi and Foà 1907: 431) (*Phylloxera*)

***OLEGIA***Shaposhnikov 1979: 734

Type species. *Aphanostigma ulmifoliae*Aoki 1973, by original designation

Etymology. Oleg (Vasilyevich Kovalev) [Russian entomologist] + -ia

Gender. Feminine

***ulmifoliae*** (Aoki 1973: 144) (*Aphanostigma*)

***PHYLLOXERA***Boyer de Fonscolombe 1834: 222

Type species. *Phylloxera quercus*Boyer de Fonscolombe 1834, by original monotypy

Etymology. Greek phýllon ‘leaf’ + Greek xērós ‘dry’

Gender. Feminine

=*ACANTHAPHIS*Del Guercio 1908: 156,157

Type species. *Phylloxera corticalis*Kaltenbach 1867, by original designation

Etymology. Greek ákantha ‘thorn’ + *Aphis* [Hemiptera: Aphididae]

Gender. Feminine

Note. Junior objective synonym of *Moritziella*Börner 1908b

=*DACTYLOSPHAERA*Shimer 1867: 290

Type species. *Dactylosphaera globosa*Shimer 1867, by original monotypy

Etymology. Greek dáktylos ‘finger’ + Greek sphaîra ‘ball’

Gender. Feminine

=*EUPHYLLOXERA*Del Guercio 1908: 155,156

Type species. *Phylloxera foveola*Pergande 1904, by original designation

Etymology. Greek eû ‘truly’ + *Phylloxera*

Gender. Feminine

=*HYSTRICHIELLA*Börner 1908b: 609

Type species. *Phylloxera spinulosa*Targioni Tozzetti 1875, by original designation

Etymology. Greek hýstrix ‘porcupine’ + -i + -ella [diminutive suffix]

Gender. Feminine

Note. Described as subgenus of *Phylloxera*Boyer de Fonscolombe 1834

=*MICRACANTHAPHIS* Grassi in Grassi et al. 1912: 48

Type species. *Vacuna coccinea*von Heyden 1837, by original designation

Etymology. Greek mikrós ‘small’ + *Acanthaphis*

Gender. Feminine

=*MORITZIELLA*Börner 1908b: 608

Type species. *Phylloxera corticalis*Kaltenbach 1867, by original designation

Etymology. (Julius) Moritz [German entomologist] + -i + ella [diminutive suffix]

Gender. Feminine

=*NOTABILIA*Mordvilko 1909: 362

Type species. *Phylloxera notabilis*Pergande 1904, by original designation

Etymology. Latin notabilis ‘remarkable, sizeable’, inflected in the neuter plural

Gender. Neuter

=*PARAMORITZIELLA* Grassi in Grassi et al. 1912: 13

Type species. *Phylloxera caryaefoliae*Fitch 1856, by original designation

Etymology. Greek pará ‘beside’ + *Moritziella*

Gender. Feminine

=*PARAPERGANDEA*Börner 1930: 160

Type species. *Phylloxera caryaevenae*Fitch 1856, by original designation

Etymology. Greek pará ‘beside’ + *Pergandea*

Gender. Feminine

=*PARAPHYLLOXERA* Grassi in Grassi et al. 1912: 11,60

Type species. *Vacuna glabra*von Heyden 1837, by original designation

Etymology. Greek pará ‘beside’ + *Phylloxera*

Gender. Feminine

=*PARTHENOPHYLLOXERA* Grassi in Grassi et al. 1912: 11,62

Type species. *Parthenophylloxera ilicis*Grassi 1912, by original designation

Etymology. Greek parthénos ‘girl, virgin’ + *Phylloxera*

Gender. Feminine

=*PERGANDEA*Börner 1908b: 610

Type species. *Dactylosphaera conica*Shimer 1869, by original designation

Etymology. (Theodore) Pergande [American entomologist] + -a

Gender. Feminine

Note. Junior homonym of *Pergandea*Ashmead 1905: 382 (Hymenoptera). Described as subgenus of *Dactylosphaera*Shimer 1867

=*PHYLLOXERELLA* Grassi in Grassi et al. 1912: 11,54

Type species. *Phylloxerella confusa*Grassi 1912, by original designation

Etymology. *Phylloxera* + -ella [diminutive suffix]

Gender. Feminine

=*PHYLLOXEROIDES* Grassi in Grassi et al. 1912: 11,48

Type species. *Phylloxera italica*Grassi 1912, by original designation

Etymology. *Phylloxera* + Greek –ō(i)dēs ‘resembling’

Gender. Masculine

=*PSYLLOPTERA*Ferrari 1872: 85

Type species. *Psylloptera quercina*Ferrari 1872, by original monotypy

Etymology. *Psylla* [Hemiptera: Psyllidae] + Greek pterá ‘wings’

Gender. Feminine

=*RHANIS*von Heyden 1837: 289

Type species. None

Etymology. Greek rhanís ‘drop (of a liquid)’

Gender. Feminine

Note. Unavailable, described in synonymy with *Vacuna*von Heyden 1837. Junior homonym of *Rhanis*Dejean 1836: 440 (Coleoptera)

=*TROITZKYA*Börner 1930: 160

Type species. *Dactylosphaera caryaesemen*Walsh 1867, by original designation

Etymology. (Nikolay Nikolaevich) Troitzky [Russian entomologist] + -a

Gender. Feminine

=*VACUNA*von Heyden 1837: 289

Type species. *Vacuna coccinea*von Heyden 1837, by original monotypy

Etymology. Latin Vacuna [minor goddess of ancient Italy]

Gender. Feminine

=*XEROPHYLLA*Walsh 1867: 283

Type species. *Pemphigus caryaecaulis*Fitch 1855, by subsequent designation (Börner 1930: 159)

Etymology. Greek xērós ‘dry’ + Greek phýllon ‘leaf’

Gender. Feminine

***caryaeavellana***Riley 1880: 230 (*Phylloxera*)

***caryaecaulis*** (Fitch 1855: 859) (*Pemphigus*)

=*caryaemagna* (Shimer 1869: 391) (*Dactylosphaera*)

***caryaefallax***Riley 1874a: 1387 (*Phylloxera*)

***caryaefoliae***Fitch 1856: 446 (*Phylloxera*)

***caryaeglobuli***Walsh 1863: 309 (*Phylloxera*)

=*hemisphericum* (Shimer 1869: 387) (*Dactylosphaera*)

***caryaegummosa***Riley 1874a: 1387 (*Phylloxera*)

***caryaepilula*** (Walsh 1867: 283) (*Xerophylla*) nomen nudum

***caryaeren***Riley 1874a: 1387 (*Phylloxera*) original spelling *caryaereniformis* but *caryaeren* in prevailing usage (ICZN Article 33.3.1)

***caryaescissa***Riley 1880: 230 (*Phylloxera*)

***caryaesemen*** (Shimer 1869: 392) (*Dactylosphaera*) specific epithet first used by Walsh (1867: 283), but not placed in combination with a genus and hence unavailable until Shimer established it as a binomen

***caryaesepta*** (Shimer 1869)

subspecies ***caryaesepta*** (Shimer 1869: 389) (*Dactylosphaera*)

subspecies ***perforans***Pergande 1904: 188,193 (variety of *Phylloxera caryaesepta* (Shimer 1869))

***caryaevenae*** (Fitch 1856: 444) (*Pemphigus*)

***castaneae*** (Haldeman 1850: 106) (*Chermes*)

***castaneivora*** (Miyazaki 1968: 400) (*Moritziella*)

***coccinea*** (von Heyden 1837: 289) (*Vacuna*)

=*escorialensis*Lichtenstein 1876: 130 (*Phylloxera*) nomen nudum

=*globifera* (von Heyden 1837: 289) (*Rhanis*) unavailable, described in synonymy with *Vacuna coccinea*von Heyden 1837

=*rutila*Dreyfus 1889: 95 (*Phylloxera*)

***confusa*** Grassi in Grassi et al. 1912: 54 (*Phylloxera*)

***conica*** (Shimer 1869: 390) (*Dactylosphaera*)

***corticalis***Kaltenbach 1867: 44 (*Phylloxera*)

=*iberica*Staroselsky 1892: 177 (*Phylloxera*)

=*lichtensteinii*Balbiani 1874: 645 (*Phylloxera*)

***davidsoni***Duncan 1922: 271 (*Phylloxera*)

***deplanata***Pergande 1904: 188,205 (*Phylloxera*)

***depressa*** (Shimer 1869: 390) (*Dactylosphaera*)

***devastatrix***Pergande 1904: 243,248 (*Phylloxera*)

***foae***Börner 1909a: 26 (*Phylloxera*)

***foveata*** (Shimer 1869: 393) (*Dactylosphaera*)

***foveola***Pergande 1904: 188,200 (*Phylloxera*)

*fraxini*Stebbins 1910: 46 (*Phylloxera*) nomen dubium, only the gall was described and it is probably not a phylloxerid

***georgiana***Pergande 1904: 243,249 (*Phylloxera*)

***glabra*** (von Heyden 1837: 291) (*Vacuna*)

=*punctata*Lichtenstein 1874b:CCI (*Phylloxera*) original name *bipunctatum* but *punctata* in prevailing usage (ICZN Article 33.3.1)

***globosa*** (Shimer 1867)

subspecies ***coniferum*** (Shimer 1869: 397) (*Dactylosphaera*)

subspecies ***globosa*** (Shimer 1867: 2) (*Dactylosphaera*)

***ilicis*** (Grassi in Grassi et al. 1912: 62) (*Parthenophylloxera*)

***intermedia***Pergande 1904: 188,189 (*Phylloxera*)

***italica*** (Grassi in Grassi et al. 1912: 48) (*Phylloxeroides*)

***kunugi***Shinji 1943: 2 (*Phylloxera*)

***minima*** (Shimer 1869: 391) (*Dactylosphaera*)

***notabilis***Pergande 1904: 217,235 (*Phylloxera*)

***perniciosa***Pergande 1904: 244,251 (*Phylloxera*)

***picta***Pergande 1904: 188,197 (*Phylloxera*)

***pilosula***Pergande 1904: 188,203 (*Phylloxera*)

***querceti***Pergande 1904: 263 (*Phylloxera*)

***quercina*** (Ferrari 1872: 85) (*Psylloptera*)

=*spinulosa*Targioni Tozzetti 1875: 308 (*Phylloxera*)

***quercus***Boyer de Fonscolombe 1834: 223 (*Phylloxera*)

=*florentina*Targioni Tozzetti 1875: 287 (*Phylloxera*)

=*scutifera*Signoret 1867: 303 (*Phylloxera*) nomen dubium; Signoret (1867) wrote he was unable to find significant differences between this species and *Phylloxera quercus* Boyer de Fonscolombe except that *scutifera* was “slightly larger and darker”; he also drew a scale-like structure (Plate 7, Figure 6) that is not of phylloxerid origin, suggesting his description included a mixture of species

=*signoreti*Targioni Tozzetti 1875: 302 (*Phylloxera*)

***reticulata***Duncan 1922: 271 (*Phylloxera*)

***rileyi***Riley 1874b: 64 (*Phylloxera*)

***rimosalis***Pergande 1904: 216,217 (*Phylloxera*)

***russellae***Stoetzel 1981: 128 (*Phylloxera*)

***similans***Duncan 1922: 272 (*Phylloxera*)

***spinifera***Pergande 1904: 261 (*Phylloxera*)

***spinosa*** (Shimer 1869: 397) (*Dactylosphaera*)

***spinuloides***Pergande 1904: 243 (*Phylloxera*)

***stanfordiana***Ferris 1919: 103 (*Phylloxera*)

***stellata***Duncan 1922: 269 (*Phylloxera*)

***subelliptica*** (Shimer 1869: 389) (*Dactylosphaera*)

***symmetrica***Pergande 1904

subspecies ***purpurea***Pergande 1904: 232 (variety of *Phylloxera symmetrica*Pergande 1904)

subspecies ***symmetrica***Pergande 1904: 218,230 (*Phylloxera*)

subspecies ***vasculosa***Pergande 1904: 233 (variety of *Phylloxera symmetrica*Pergande 1904)

***texana***Stoetzel 1981: 141 (*Phylloxera*)

***tuberculifera***Duncan 1922: 272 (*Phylloxera*)

Subfamily **PHYLLOXERININAE**Börner 1908b: 607

Original spelling. Phylloxerinini

Type genus. *Phylloxerina*Börner 1908a

***PHYLLOXERINA***Börner 1908a: 94

Type species. *Phylloxera salicis*Lichtenstein 1884, by original monotypy

Etymology. *Phylloxera* + Latin -ina ‘in relation to’

Gender. Feminine

=*GUERCIOJA*Mordvilko 1909: 361

Type species. *Chermes populi*Del Guercio 1900, by original designation

Etymology. (Giacomo Del) Guercio [Italian entomologist] + -ja

Gender. Feminine

=*LAUFFERELLA*Lindinger 1933: 32

Type species. *Chermes populi*Del Guercio 1900, inherited from replaced name

Etymology. (Jorge) Lauffer [German entomologist] + -ella [diminutive suffix]

Gender. Feminine

Note. Replacement name for *Pseudochermes*Bonfigli 1909. Junior objective synonym of *Guercioja*Mordvilko 1909

=*PSEUDOCHERMES*Bonfigli 1909: 398

Type species. *Chermes populi*Del Guercio 1900, by original monotypy

Etymology. Greek pseudo- ‘untrue’ + *Chermes* [Hemiptera]

Gender. Masculine

Note. Junior homonym of *Pseudochermes* Nitsche in Judeich and Nitsche 1895: 1248 (Hemiptera: Cryptococcidae). Replaced by *Lauferella*Lindinger 1933

***capreae***Börner 1942: 265 (*Phylloxerina*)

***daphnoidis***Iglisch 1965: 424 (*Phylloxerina*)

***moniliferae*** (Börner 1931: 696) (*Guercioja*) new name for *Chermes populi*Gillette 1914; possible synonym of *Phylloxerina popularia* (Pergande)

=*populi* (Gillette 1914: 269) (*Chermes*) junior primary homonym of *Phylloxerina populi* (Del Guercio 1900)

***nyssae*** (Pergande 1904: 269) (*Phylloxera*)

***popularia*** (Pergande 1904: 266) (*Phylloxera*)

***populi*** (Del Guercio 1900: 81,83) (*Chermes*)

***prolifera*** (Oestlund 1887: 16) (*Phylloxera*)

***salicis*** (Lichtenstein 1884: 616) (*Phylloxera*)

***salicola*** (Pergande 1904: 267) (*Phylloxera*)

### Index of genus-group and species-group names

*ACANTHAPHIS* Del Guercio 1908 – synonym of *Phylloxera*

***ACANTHOCHERMES*** Kollar 1848 – Phylloxerinae, Acanthochermesini

***APHANOSTIGMA*** Börner 1909b – Phylloxerinae, Phylloxerini

*balbianii* Lichtenstein 1874a – synonym of *Acanthochermes quercus*

*bipunctata* Lichtenstein 1874b – see *punctata*

*BOERNERIA* Grassi and Foà 1908 – synonym of *Foaiella*

***capreae*** Börner 1942 – *Phylloxerina*

***caryaeavellana*** Riley 1880 – *Phylloxera*

***caryaecaulis*** Fitch 1855 – *Phylloxera*

***caryaefallax*** Riley 1874a – *Phylloxera*

***caryaefoliae*** Fitch 1856 – *Phylloxera*

***caryaeglobuli*** Walsh 1863 – *Phylloxera*

***caryaegummosa*** Riley 1874a – *Phylloxera*

***caryaemagna*** Shimer 1869 – synonym of *Phylloxera caryaecaulis*

***caryaepilula*** Walsh 1867 – *Phylloxera*

***caryaeren*** Riley 1874a – *Phylloxera*

***caryaereniformis*** Riley 1874a – see *caryaeren*

***caryaescissa*** Riley 1880 – *Phylloxera*

***caryaesemen*** Shimer 1869 – *Phylloxera*

***caryaesepta*** Shimer 1869 – *Phylloxera*

***caryaevenae*** Fitch 1856 – *Phylloxera*

***castaneae*** Haldeman 1850 – *Phylloxera*

***castaneivora*** Miyazaki 1968 – *Phylloxera*

*CINACIUM* Kishida 1924 – synonym of *Aphanostigma*

***coccinea*** von Heyden 1837 – *Phylloxera*

***confusa*** Grassi in Grassi et al. 1912 – *Phylloxera*

***conica*** Shimer 1869 – *Phylloxera*

***coniferum*** Shimer 1869 – subspecies of *Phylloxera globosa*

***corticalis*** Kaltenbach 1867 – *Phylloxera*

*DACTYLOSPHAERA* Shimer 1867 – synonym of *Phylloxera*

***DAKTULOSPHAIRA*** Shimer 1866 – Phylloxerinae, Phylloxerini

***danesii*** Grassi and Foà 1907 – *Foaiella*

***daphnoidis*** Iglisch 1965 – *Phylloxerina*

***davidsoni*** Duncan 1922 – *Phylloxera*

***deplanata*** Pergande 1904 – *Phylloxera*

***depressa*** Shimer 1869 – *Phylloxera*

***devastatrix*** Pergande 1904 – *Phylloxera*

*escorialensis* Lichtenstein 1876 – synonym of *Phylloxera coccinea*

*EUPHYLLOXERA* Del Guercio 1908 – synonym of *Phylloxera*

*florentina* Targioni Tozzetti 1875 – synonym of *Phylloxera quercus*

***foae*** Börner 1909a – *Phylloxera*

***FOAIELLA*** Börner 1909b – Phylloxerinae, Phylloxerini

***foveata*** Shimer 1869 – *Phylloxera*

***foveola*** Pergande 1904 – *Phylloxera*

*fraxini* Stebbins 1910 – *Phylloxera*

***georgiana*** Pergande 1904 – *Phylloxera*

***glabra*** von Heyden 1837 – *Phylloxera*

*globifera* von Heyden 1837 – synonym of *Phylloxera coccinea*

***globosa*** Shimer 1867 – *Phylloxera*

*GUERCIOJA* Mordvilko 1909 – synonym of *Phylloxerina*

*hemisphericum* Shimer 1869 – synonym of *Phylloxera caryaeglobuli*

*HYSTRICHIELLA* Börner 1908b – synonym of *Phylloxera*

***iaksuiense*** Kishida 1924 – *Aphanostigma*

*iberica* Staroselsky 1892 – synonym of *Phylloxera corticalis*

***ilicis*** Grassi in Grassi et al. 1912 – *Phylloxera*

***intermedia*** Pergande 1904 – *Phylloxera*

***italica*** Grassi in Grassi et al. 1912 – *Phylloxera*

***kunugi*** Shinji 1943 – *Phylloxera*

*LAUFFERELLA* Lindinger 1933– synonym of *Phylloxerina*

*lichtensteinii* Balbiani 1874 – synonym of *Phylloxera corticalis*

*MICRACANTHAPHIS* Grassi in Grassi et al. 1912 – synonym of *Phylloxera*

***minima*** Shimer 1869 – *Phylloxera*

***moniliferae*** Börner 1931 – *Phylloxerina*

*MORITZIELLA* Börner 1908b – synonym *Phylloxera*

*NOTABILIA* Mordvilko 1909 – synonym of *Phylloxera*

***notabilis*** Pergande 1904 – *Phylloxera*

***nyssae*** Pergande 1904 – *Phylloxerina*

***OLEGIA*** Shaposhnikov 1979 – Phylloxerinae, Phylloxerini

*PARAMORITZIELLA* Grassi in Grassi et al. 1912 – synonym of *Phylloxera*

*PARAPERGANDEA* Börner 1930 – synonym of *Phylloxera*

*PARAPHYLLOXERA* Grassi in Grassi et al. 1912 – synonym of *Phylloxera*

*PARTHENOPHYLLOXERA* Grassi in Grassi et al. 1912 – synonym of *Phylloxera*

*pemphigoides* Donnadieu 1887 – synonym of *Daktulosphaira vitifoliae*

***perforans*** Pergande 1904 – subspecies of *Phylloxera caryaesepta*

*PERGANDEA* Börner 1908b – synonym of *Phylloxera*

*PERITYMBIA* Westwood 1869 – synonym of *Daktulosphaira*

***perniciosa*** Pergande 1904 – *Phylloxera*

***pervastatrix*** Börner 1910 – synonym of *Daktulosphaira vitifoliae*

***PHYLLOXERA*** Boyer de Fonscolombe 1834 – Phylloxerinae, Phylloxerini

*PHYLLOXERELLA* Grassi in Grassi et al. 1912 – synonym of *Phylloxera*

***PHYLLOXERINA*** Börner 1908a – Phylloxerininae

*PHYLLOXEROIDES* Grassi in Grassi et al. 1912 – synonym of *Phylloxera*

***picta*** Pergande 1904 – *Phylloxera*

***pilosula*** Pergande 1904 – *Phylloxera*

***piri*** Cholodkovsky 1904 – *Aphanostigma*

***popularia*** Pergande 1904 – *Phylloxerina*

***populi*** Del Guercio 1900 – *Phylloxerina*

*populi* Gillette 1914 – synonym of *Phylloxerina moniliferae*

***prolifera*** Oestlund 1887 – *Phylloxerina*

*PSEUDOCHERMES* Bonfigli 1909 – synonym of *Phylloxerina*

*PSYLLOPTERA* Ferrari 1872 – synonym of *Phylloxera*

*punctata* Lichtenstein 1874b – synonym of *Phylloxera glabra*

***purpurea*** Pergande 1904 – subspecies of *Phylloxera symmetrica*

***querceti*** Pergande 1904 – *Phylloxera*

***quercina*** Ferrari 1872 – *Phylloxera*

***quercus*** Boyer de Fonscolombe 1834 – *Phylloxera*

***quercus*** Kollar 1848 – *Acanthochermes*

***reticulata*** Duncan 1922 – *Phylloxera*

*RHANIS* von Heyden 1837 – synonym of *Phylloxera*

*RHIZAPHIS* Planchon in Bazille et al. 1868 – synonym of *Daktulosphaira*

*RHIZOCERA* Despeissis 1896 – synonym of *Daktulosphaira*

***rileyi*** Riley 1874b – *Phylloxera*

***rimosalis*** Pergande 1904 – *Phylloxera*

***russellae*** Stoetzel 1981 – *Phylloxera*

*rutila* Dreyfus 1889 – synonym of *Phylloxera coccinea*

***salicis*** Lichtenstein 1884 – *Phylloxerina*

***salicola*** Pergande 1904 – *Phylloxerina*

*scutifera* Signoret 1867 – synonym of *Phylloxera quercus*

*signoreti* Targioni Tozzetti 1875 – synonym of *Phylloxera quercus*

***similans*** Duncan 1922 – *Phylloxera*

***similiquercus*** Jiang et al. 2009 – *Acanthochermes*

***spinifera*** Pergande 1904 – *Phylloxera*

***spinosa*** Shimer 1869 – *Phylloxera*

***spinuloides*** Pergande 1904 – *Phylloxera*

*spinulosa* Targioni Tozzetti 1875 –synonym of *Phylloxera quercina*

***stanfordiana*** Ferris 1919 – *Phylloxera*

***stellata*** Duncan 1922 – *Phylloxera*

***subelliptica*** Shimer 1869 – *Phylloxera*

***symmetrica*** Pergande 1904 – *Phylloxera*

***texana*** Stoetzel 1981 – *Phylloxera*

*TROITZKYA* Börner 1930 – synonym of *Phylloxera*

***tuberculifera*** Duncan 1922 – *Phylloxera*

***ulmifoliae*** Aoki 1973 – *Olegia*

*VACUNA* von Heyden 1837 – synonym of *Phylloxera*

***vasculosa*** Pergande 1904 – subspecies of *Phylloxera symmetrica*

*vastatrix* Planchon in Bazille et al. 1868 – synonym of *Daktulosphaira vitifoliae*

*VITEUS* Shimer 1867 – synonym of *Daktulosphaira*

***vitifoliae*** Fitch 1855 – *Daktulosphaira*

*vitis viniferae* Theobald 1914 – synonym of *Daktulosphaira vitifoliae*

*vitisana* Westwood 1869 – synonym of *Daktulosphaira vitifoliae*

*vulpinae* Börner 1952 – synonym of *Daktulosphaira vitifoliae*

*XERAMPELUS* Del Guercio 1900 – synonym of *Daktulosphaira*

*XEROPHYLLA* Walsh 1867 – synonym of *Phylloxera*

## References

[B1] Alleweldt G Spiegel-Roy P Reisch B (1991) Grapes (*Vitis*). Acta Horticulturae 290: 291–330. doi: 10.17660/ActaHortic.1991.290.7

[B2] Aoki S (1973) A new gall-making phylloxerid on the leaves of elm in Japan (Homoptera: Aphidoidea). Kontyû 41(2): 144–147.

[B3] Ashmead WH (1905) A skeleton of a new arrangement of the families, subfamilies, Tribes and genera of the ants, or the superfamily Formicoidea. The Canadian Entomologist 37(11): 381–384. doi: 10.4039/Ent37381-11

[B4] Axelson CB (1902) Diagnosen neuer Collembolen aus Finland und angrenzenden Teilen des nordwestlichen Russlands. Meddelanden af Societas pro Fauna et Flora Fennica 28: 101–111.

[B5] Balbiani ÉG (1874) Sur la prétendue migration des Phylloxeras ailés sur les chênes à kermès. Comptes rendus hebdomadaires des Séances de l’Académie des Sciences 79: 640–645.

[B6] Bazille G Planchon JE Sahut F (1868) Sur une maladie de la vigne actuellement régnante en Provence. Comptes rendus hebdomadaires des Séances de l’Académie des Sciences 6–7: 336.

[B7] Bonfigli B (1909) Intorno ad un Fillosserinino des *Populus alba*. Atti della Reale Accademia dei Lincei Fifth Series 18(9): 397–403.

[B8] Börner C (1908a) Eine monographische Studie über die Chermiden. Arbeiten aus der Kaiserlichen Biologischen Anstalt für Land- und Forstwirtschaft 6(2): 81–320.

[B9] Börner C (1908b) Über Chermesiden I. Zur Systematik der Phylloxerinen. Zoologischer Anzeiger 33(17-18): 600–612.

[B10] Börner C (1909a) Über Chermesiden V. Die Zucht des Reblaus-Wintereies in Deutschland. Zoologischer Anzeiger 34(1): 13–29.

[B11] Börner C (1909b) Untersuchungen über die Phylloxerinen (Reblaus und verwandte Formen). Mitteilungen aus der Kaiserlichen Biologischen Anstalt für Land- und Forstwirtschaft 8: 60–72.

[B12] Börner C (1910) Die Deutsche Reblaus, eine durch Anpassung an die Europäerrebe entstandene Varietät. Self-published, Sankt Julian bei Metz, 4 pp.

[B13] Börner C (1913) Aphidoïden. Aphididen, Blattläuse. In: Reh L (Ed.) Handbuch der Pflanzenkrankheiten von Prof. Dr. Paul Sorauer, Volume 3. Paul Parey, Berlin, 654–683.

[B14] Börner C (1930) Beiträge zu einem neuen System der Blattläuse. Archiv für klassifikatorische und phylogenetische Entomologie 1(2): 115–180.

[B15] Börner C (1931) Aphidoidea, Blattläuse. Sorauer Handbuch der Pflanzenkrankheiten [offprint], 165 pp. See Nieto Nafría et al. 2007: 54.

[B16] Börner C (1942) Weitere neue europäische Blattlausarten. Veröffentlichungen aus dem Deutschen Kolonial- und Übersee-Museum in Bremen 3(3): 259–266.

[B17] Börner C (1952) Europae centralis Aphides. Die Blattläuse Mitteleuropas. Namen, Synonyme, Wirtspflanzen, Generationszyklen. Mitteilungen der Thüringischen Botanischen Gesellschaft supplement 3: 1–259.

[B18] Boyer de Fonscolombe ÉLJH (1834) Notice sur les genres d’Hymenoptères *Lithurgus* et *Phylloxera*. Annales de la Société Entomologique de France 3: 219–224.

[B19] Cholodkovsky NA (1904) Aphidologische Mitteilungen. Zoologischer Anzeiger 27(4): 118–119.

[B20] Cortés Gabaudan F Nieto Nafría JM Favret C Barbagallo S Sano M Stekolshchikov AV (2011) Etymology and gender of genus-group names. In: Nieto Nafría JM Favret C (Eds) Registers of Family-Group and Genus-Group Taxa of Aphidoidea (HemipteraSternorrhyncha). Universidad de León, León, 405–463.

[B21] Dejean PFMA (1836) Catalogue des Coléoptères de la collection de M. le Comte Dejean. [Livraison 5]. Méquignon-Marvis, Paris, 361–443.

[B22] Del Guercio G (1900) Prospetto dell’afidofauna italica. Nuove Relazioni Intorno ai Lavori della Regia Stazione di Entomologia Agraria di Firenze Serie Prima 2: 1–236.

[B23] Del Guercio G (1908) Sulla sistematica e sulla biologia dei fillosserini con un cenno intorno ad un nuovo metodo di disinfezione per le viti americane ed Europee. Privately published, Firenze, 153–188.

[B24] Despeissis A (1896) *Phylloxera* of the vine. The Agricultural Gazette of New South Wales 6(1): 13–29.

[B25] Donnadieu AL (1887) Sur les deux espèces de *Phylloxera* de la vigne. Comptes rendus hebdomadaires des Séances de l’Académie des Sciences 104: 1246–1249.

[B26] Dreyfus LT (1889) Neue Beobachtungen bei den Gattungen *Chermes* L. und *Phylloxera* Boyer de Fonsc. Zoologischer Anzeiger 12(300): 91–99.

[B27] Duncan CD (1922) The North American species of *Phylloxera* infesting oak and chestnut (Hemiptera: Phylloxeridae). The Canadian Entomologist 54(12): 267–276. doi: 10.4039/Ent54267-12

[B28] Eastop VF Hille Ris Lambers D (1976) Survey of the World’s Aphids. Dr. W. Junk, The Hague, 573 pp.

[B29] Fahrentrapp J Müller L Schumacher P (2015) Is there need for leaf-galling grape phylloxera control? Presence and distribution of *Dactulosphaira vitifoliae* in Swiss vineyards. International Journal of Pest Management 61(4): 340–345. doi: 10.1080/09670874.2015.1067734

[B30] Favret C Miller GL Nieto Nafría JM Cortés Gabaudan F (2008) [2007] Catalog of the aphid genera described from the New World. Transactions of the American Entomological Society 133(3–4): 363–412.

[B31] Favret C Miller GL Nieto Nafría JM Cortés Gabaudan F (2009) [2008]. Corrections and additions to the catalog of the aphid genera described from the New World. Transactions of the American Entomological Society 134(3–4): 275–282.

[B32] Favret C Havill NP Miller GL Sano M Victor B (2015) Catalog of the adelgids of the world (Hemiptera, Adelgidae). ZooKeys 534: 35–54. doi: 10.3897/zookeys.534.6456 10.3897/zookeys.534.6456 PMC4669935 26668546

[B33] Ferrari PM (1872) Aphididae Liguriae. Annali del Museo Civico di Storia Naturale di Genova 2: 49–85.

[B34] Ferris GF (1919) Two species of *Phylloxera* from California (Hemiptera; Aphidae). Entomological News 30(4): 103–105.

[B35] Fitch A (1855) [1854] Report on the noxious, beneficial, and other insects of the state of New York. Transactions of the New York State Agricultural Society 14: 691–880.

[B36] Fitch A (1856) Third report on the noxious and other insects of the state of New York. Transactions of the New York State Agricultural Society 16: 315–490.

[B37] Gillette CP (1914) Two Colorado plant lice (Hemip.-Homop.). Entomological News 25(6): 269–275.

[B38] Grassi GB Foà A (1907) Inaspettata scoperta di una fillossera sulle radici della quercia. Atti della Reale Accademia dei Lincei 16(7): 429–431.

[B39] Grassi GB Foà A (1908) Sulla classificazione delle Fillossere. Atti della Reale Accademia dei Lincei Fifth Series 17(12): 683–690.

[B40] Grassi GB Foà A Grandori R Bonfigli B Topi M (1912) Contributo alla Conoscenza delle fillosserine ed in particolare della fillossera della vite. Ministero d’Agricoltura, Industria e Commercio, Rome, 459 pp.

[B41] Haldeman SS (1850) On four new species of Hemiptera of the genera *Ploiaria*, *Chermes*, and *Aleurodes*, and two new Hymenoptera parasitic in the last named genus. American Journal of Science and Arts Second series 9: 108–111.

[B42] Heie OE Peñalver E (1999) *Palaeophylloxera* nov. gen., the first fossil specimen of the family Phylloxeridae (Hemiptera, Phylloxeroidea); Lower Miocene of Spain. Geobios 32(4): 593–597. doi: 10.1016/S0016-6995(99)80008-4

[B43] Heie OE Wegierek P (2009) A classification of the Aphidomorpha (Hemiptera Sternorrhyncha) under consideration of the fossil taxa. Redia 92: 69–77.

[B44] Heie OE Wegierek P (2011) A list of fossil aphids (Hemiptera, Sternorrhyncha, Aphidomorpha). Monographs of the Upper Silesian Museum 6: 1–82.

[B45] Herrich-Schaeffer D (1854) Vorwort des Herausgebers. In Koch CL, Die Pflanzenläuse Aphiden getreu nach dem Leben abgebildet und beschrieben, Volume 1 J.L. Lotzbeck, Nürnberg, III–VIII.

[B46] Iglisch I (1965) Die Biologie und Morphologie der *Phylloxerina*-Arten Deutschlands (Zwergläuse [Aphidoidea: Phylloxeridae]). Zeitschrift für Angewandte Zoologie 52: 399–474.

[B47] International Commission on Zoological Nomenclature (ICZN) (1999) International Code of Zoological Nomenclature, Fourth Edition. International Trust for Zoological Nomenclature, London, 306 pp.

[B48] Jiang Liyun Huang Xiaolei Qiao Gexia (2009) Review of the Chinese new record genus *Acanthochermes* Kollar (Hemiptera: Phylloxeridae), with a description of one new species. Pan-Pacific Entomologist 85(2): 43–50. doi: 10.3956/2007-20.1

[B49] Judeich JF Nitsche H (1895) Lehrbuch der Mitteleuropäischen Forstindektenkunde, Volume 2 Paul Parey, Berlin, 737–1421.

[B50] Kaltenbach JH (1867) Die deutschen Phytophagen aus der Klasse der Insekten. Verhandlungen des naturhistorischen Vereines der preussischen Rheinlande und Westphalens 24: 21–117.

[B51] Kirk TW (1897) Leaflets for gardeners and fruitgrowers No. 20 - *Phylloxera*. Otago Witness 2252: 7–8.

[B52] Kishida K (1924) A new aphid injurious to the pear in Japan. Dobutsugaku Zasshi (Zoological Magazine) 36(433): 472–474.

[B53] Kleeberg H Hummel E (2001) Experiences with Neemazal™ -T/S in 1994-2000. In: Metspalu L Mitt S (Eds) Practice Oriented Results on the Use of Plant Extracts and Pheromones in Pest Control. Proceedings of the international workshop, 24-25 January 2001 Tartu, Estonia, 37–45.

[B54] Kollar C (1848) Beitrag zur Entwicklungsgeschichte eines neuen, blattlausartigen Insektes: *Acanthochermes quercus*. Sitzungsberichte der Mathematisch-Naturwissenschaftlichen Classe der Kaiserlichen Akademie der Wissenschaften 1: 191–194.

[B55] Lichtenstein J (1874a) Observations, à propos de la communication récente de M. Balbiani, sur les diverses espèces connues du genre *Phylloxera*. Comptes rendus hebdomadaires des Séances de l’Académie des Sciences 79: 781–783.

[B56] Lichtenstein J (1874b) [No title]. Annales de la Société Entomologique de France, Cinquième série 4: CXCVIII–CCI.

[B57] Lichtenstein J (1876) Notes pour servir à l’histoire des insectes du genre *Phylloxera*. Annales Agronomiques 2: 127–138.

[B58] Lichtenstein J (1884) Sur un nouvel insecte du genre *Phylloxera* (*Phylloxera salicis*, Lichtenstein). Comptes rendus hebdomadaires des Séances de l’Académie des Sciences 99: 616–617.

[B59] Lindinger L (1933) Beitrage zur Kenntnis der Schildlause. Entomologische Rundschau 50: 31–32.

[B60] Loxdale HD (2008) The nature and reality of the aphid clone: genetic variation, adaptation and evolution. Agricultural and Forest Entomology 10(2): 81–90. doi: 10.1111/j.1461-9563.2008.00364.x

[B61] Miyazaki M (1968) A new species of the genus *Moritziella* Börner from Japan (Homoptera: Phylloxeridae). Kontyû 36(4): 400–402.

[B62] Mordvilko AK (1909) [1908] Tableaux pour servir à la détermination des groupes et des genres des Aphididae Passerini. Ezegodnik Zoologiceskago Muzeja Imperatorskoj Nauk Sankt Peterburg 13: 353–384.

[B63] Nieto Nafría JM Pérez Hidalgo N Mier Durante MP (2007) New synonyms and several nomenclatural clarifications on family-group names in the Aphididae (Hemiptera Sternorrhyncha). Zootaxa 1629: 51–55.

[B64] Nieto Nafría JM Favret C Akimoto S Barbagallo S Chakrabarti S Mier Durante MP Miller GL Qiao GX Sano M Pérez Hidalgo N Stekolshchikov AV Wegierek P (2011) Register of genus-group taxa of Aphidoidea. In: Nieto Nafría JM Favret C (Eds) Registers of Family-Group and Genus-Group Taxa of Aphidoidea (HemipteraSternorrhyncha). Universidad de León, León, 81–404.

[B65] Oestlund OW (1887) Synopsis of the Aphididae of Minnesota. Bulletin of the Geological and Natural History Survey of Minnesota 4: 1–100.

[B66] Pergande T (1904) North American Phylloxerinae affecting *Hicoria* (Carya) and other trees. Proceedings of the Davenport Academy of Sciences 9: 185–271. doi: 10.5962/bhl.title.54119

[B67] Remaudière G Remaudière M (1997) Catalogue of the World’s Aphididae. INRA, Paris, 473 pp.

[B68] Riley CV (1874a) Les espèces américaines du genre *Phylloxera*. Comptes rendus hebdomadaires des Séances de l’Académie des Sciences 79(24): 1384–1388.

[B69] Riley CV (1874b) [1873] Sixth annual report of the noxious, beneficial and other insects of the state of Missouri. Annual Report of the State Board of Agriculture of Missouri, 169 pp.

[B70] Riley CV (1880) New hickory galls made by *Phylloxera*. The American Entomologist v. 3, 2nd ser v. 1(9): 230.

[B71] Russell LM (1975) [1974] *Daktulosphaira vitifoliae* (Fitch), the correct name of the grape phylloxeran (Hemiptera: Homoptera: Phylloxeridae). Journal of the Washington Academy of Sciences 64(4): 303–308.

[B72] Shaposhnikov GK (1979) Oligomerization, polymerization and organization of morphological structures in the evolution of aphids (Homoptera, Aphidinea). Entomologicheskoe Obozrenie 58(4): 716–741.

[B73] Shimer H (1866) “Grape leaf louse.” – *Daktulosphaira vitifoliae*, continued from page 290. The Prairie Farmer 18: 365.

[B74] Shimer H (1867) On a new genus in Homoptera,–(Section Monomera). Proceedings of the Academy of Natural Sciences of Philadelphia 19: 2–11.

[B75] Shimer H (1869) [1868] A summers study of hickory galls, with descriptions of supposed new insects bred therefrom. Transactions of the American Entomological Society 2(1): 386–398.

[B76] Shinji GO (1943) Description of a new species of *Phylloxera*. Insect World 47(1): 2–3.

[B77] Signoret VA (1867) Études sur le genre *Phylloxera* de Fonscolombe. Annales de la Société Entomologique de France Fourth Series 7: 297–304.

[B78] Staroselsky AV (1892) On a new species of oak *Phylloxera*. Proceedings of the laboratory at the Sakara nursery of American vines. Annex to the Report of the Caucasian *Phylloxera* Committee for the year 1891, Year 1: 177–187.

[B79] Stebbins FA (1910) [1909] Insect galls of Springfield, Massachusetts, and vicinity. Springfield Museum of Natural History Bulletin 2: 1–139.

[B80] Stoetzel MB (1981) Two new species of *Phylloxera* (Phylloxeridae: Homoptera) on pecan. Journal of the Georgia Entomological Society 16(2): 127–144.

[B81] Stoetzel MB (1985) Host alternation: a newly discovered attribute of the Phylloxeridae (Homoptera: Aphidoidea). Proceedings of the Entomological Society of Washington 87(2): 265–268.

[B82] Targioni Tozzetti A (1875) Del pidocchio della Fillossera della vite e delle specie del genere *Philloxera* in Europa e in America. Bullettino della Società entomologica italiana 7(4): 266–319.

[B83] Tecchio MA Paioli-Pires EJ Terra MM Junqueira Teixeira LA Leonel S (2007) Características físicas e acúmulo de nitrientes pelos cachos de ‘Niagara rosada’ em vinhedos na região de Jundiaí. Revista Brasileira de Fruticultura 29(3): 624–625. doi: 10.1590/S0100-29452007000300038

[B84] Theobald FV (1914) African Aphididae. Bulletin of Entomological Research 4: 313–337. doi: 10.1017/S0007485300043248

[B85] Torregrossa L Viguier D Vergnettes B Planas R (1997) Phylloxéra (*Daktylosphaira vitifoliae* Fitch) et dépérissement du vignoble: Cas des parcelles audoises à la submersion. Progrès Agricole et Viticole 114(10): 223–231.

[B86] von Heyden CHG (1837) Entomologische Beiträge. Museum Senckenbergianum Abhandlungen 2(3): 287–299.

[B87] Walsh BD (1863) [1862] On the genera of Aphidae found in the United States. Proceedings of the Entomological Society of Philadelphia 1: 294–311.

[B88] Walsh BD (1867) On the insects, coleopterous, hymenopterous and dipterous, inhabiting the galls of certain species of willow – Part 2d and last. Proceedings of the Entomological Society of Philadelphia 6: 223–288.

[B89] Wegierek P (2003) Apterous Phylloxeroidea (Hemiptera, Sternorrhyncha) from Baltic amber. Acta Zoologica Cracoviensia 46(fossil insects): 277–283.

[B90] Westwood JO (1867) On a new vine disease. Proceedings of the Ashmolean Society New Series 4: 6.

[B91] Westwood JO (1869) New vine diseases. The Gardeners’ Chronicle and Agricultural Gazette 1869(5): 109.

[B92] Westwood JO (1877) The president’s address. Proceedings of the Entomological Society of London 1877: 37–81.

[B93] Willem V (1902) Collemboles. Expédition antarctique belge. Résultats du voyage du S.Y. Belgica en 1897–1898–1899 sous le commandement de A. de Gerlache de Gomery. Rapports scientifiques. Zoologie R8: 1–19.

[B94] Wilson HF (1910) A list of the genera described as new from 1858 to 1909 in the family Aphididae. Entomological News 21(4): 147–156.

[B95] Wilson HF Vickery RA (1918) A species list of the Aphididae of the world and their recorded food plants. Transactions of the Wisconsin Academy of Sciences, Arts, and Letters 19: 22–352.

